# GenIV vaccines: bridging innovation to equity in neglected tropical diseases

**DOI:** 10.3389/fimmu.2026.1756570

**Published:** 2026-03-09

**Authors:** Marta Araújo, Dhiraj Gurjar, Nicolas Grandchamp, Bhaskar Saha, Ricardo Silvestre

**Affiliations:** 1 Life and Health Sciences Research Institute (ICVS), School of Medicine from University of Minho, Braga, Portugal; 2 ICVS/3B’s – PT Government Associate Laboratory, Braga/Guimarães, Portugal; 3 National Centre for Cell Science, Pune, India; 4 GEG-Tech, Paris, France; 5 JSPS Government Homeopathic Medical College, Ramanthapur/Hyderabad, Telangana, India

**Keywords:** fourth-generation vaccines, leishmaniasis, mRNA vaccines, neglected tropical diseases, translational vaccinology, vaccine equity

## Abstract

Recent breakthroughs in molecular vaccinology have defined a new generation of vaccines that integrate synthetic mRNA, self-amplifying RNA, and nanomaterial-based platforms. These fourth-generation vaccines offer exceptional adaptability, rapid design, and strong immunogenicity, as demonstrated during the COVID-19 pandemic. Their potential now extends to neglected tropical diseases (NTDs), where conventional vaccine strategies have failed to deliver durable protection. This review traces the evolution from whole-pathogen to precision molecular vaccines, highlighting the mechanisms, delivery systems, and translational advances that underpin the GenIV paradigm. Using leishmaniasis as a case study, we discuss how these technologies can bridge innovation and equity through technology transfer, regional manufacturing, and global collaboration. By integrating scientific, ethical, and implementation perspectives, this work outlines how next-generation vaccines can transform both epidemic preparedness and the equitable control of endemic diseases.

## 1 Introduction

Vaccination remains one of the most impactful medical interventions, preventing millions of deaths annually and reshaping the epidemiology of infectious diseases worldwide (1). Classical vaccines, including live-attenuated and inactivated formulations, were instrumental in the eradication of smallpox and the near-elimination of poliomyelitis, while subunit and toxoid vaccines further improved safety and scalability in other infections (2). Nevertheless, these traditional platforms have shown limitations against pathogens with high antigenic diversity, complex life cycles, or those requiring strong cell-mediated immunity, such as HIV, *Mycobacterium tuberculosis*, and protozoan parasites (*Leishmania* spp.) (3).

Technological advances over the past three decades have enabled the emergence of nucleic acid–based platforms, including DNA, viral vectors, and more recently mRNA vaccines, which laid the groundwork for what is now defined as the fourth generation of vaccines (GenIV). This latest generation is characterized by the integration of stabilized synthetic mRNA technologies and advanced delivery systems, such as lipid nanoparticles and other protective carriers, which safeguard RNA integrity and enhance cellular targeting and translation efficiency (4, 5). The rapid development, approval, and deployment of mRNA vaccines against SARS-CoV-2 demonstrated the feasibility of producing safe and effective vaccines in record time, catalyzing unprecedented investment in genetic platforms and nanoparticle-based delivery systems (6). These successes not only transformed the response to a global pandemic but also expanded the conceptual framework of vaccines, broadening their application to cancer immunotherapy and autoimmune modulation (7).

Despite these breakthroughs, the benefits of GenIV technologies have not yet reached many neglected tropical diseases (NTDs), which continue to cause significant morbidity and mortality in low- and middle-income countries (8). Infections such as leishmaniasis, Chagas disease, schistosomiasis, malaria, and tuberculosis still lack widely effective licensed vaccines for human use, whether due to limited commercial incentives, technical barriers to inducing durable immunity, or the complex immunobiology of the pathogens involved (9). Leveraging the adaptability and immunogenic potential of novel platforms could therefore provide new solutions for these unmet needs.

In contrast to recent reviews that address mRNA vaccine technology, NTD vaccine pipelines, or vaccine equity as largely separate themes, the present work provides an integrated GenIV vaccinology framework that connects platform design, immune mechanisms, delivery systems, translational bottlenecks, and equity-driven implementation. Specifically, we (i) situate mRNA, self-amplifying RNA, and nanoparticle-based vaccines within a four-generation model, (ii) use leishmaniasis as a mechanistically informative case study to illustrate shared challenges across NTDs, and (iii) extend the discussion to actionable strategies for technology transfer, regional manufacturing, and regulatory readiness in endemic regions. This synthesis aims to bridge innovation and access, providing a translational roadmap for deploying fourth-generation platforms against multiple NTDs. By integrating these components into a single translational narrative, we aim to offer a practical roadmap for how GenIV platforms can be developed and deployed for NTDs beyond proof-of-concept preclinical studies.

## 2 From pathogen-based to platform-based vaccines: The four generations

This section focuses on the biological and immunological principles underlying fourth-generation vaccine platforms, highlighting how mRNA and nanoparticle-based designs enable precise antigen expression, innate immune activation, and adaptive immune programming. The conceptual shift from whole-pathogen vaccines to rationally designed molecular and synthetic platforms illustrates both the successes and the persistent challenges in eliciting durable and protective immunity, particularly against complex intracellular parasites. NTDs, including leishmaniasis, Chagas disease, and schistosomiasis, exemplify the translational gap: while the first and second generations have been transformative for viral and bacterial diseases, they have not yet produced a licensed human vaccine for these protozoan and helminth infections (10). An exception is the RTS, S/AS01 vaccine, which has been recommended by the WHO for malaria prevention in children aged 6 weeks to 17 months, although it requires four doses and provides only moderate and time-limited protection (*Malaria Vaccines (RTS,S and R21)*, n.d.) (11). Throughout this section, leishmaniasis is used primarily as an illustrative case study, while examples from other NTDs are incorporated to maintain broader relevance.

### 2.1 First-generation vaccines: live-attenuated and inactivated pathogens

First-generation vaccines employ whole organisms, either attenuated to reduce virulence or chemically/physically inactivated, to mimic natural infection. Their major immunological strength is the induction of broad and long-lived immunity, engaging both humoral and cellular compartments. The yellow fever vaccine (17D) and smallpox vaccine are iconic successes of this class (1). For NTDs, however, pathogen complexity and safety concerns limited their use. In leishmaniasis, leishmanization, the deliberate inoculation of live *L. major*, was practiced for centuries in parts of the Middle East and Central Asia. While it conferred durable protection against cutaneous disease, it carried unacceptable risks of chronic lesions, uncontrolled dissemination, and contraindications in immunocompromised individuals (12). Attempts to produce killed *Leishmania* vaccines (e.g., autoclaved or freeze-thawed promastigotes combined with adjuvants such as BCG) showed immunogenicity and even partial protection in experimental settings [5]. Yet, large-scale field trials in humans failed to reproduce efficacy, reflecting variability in parasite strains, manufacturing inconsistency, and the inability of inactivated parasites to induce robust T cell memory. Similar challenges were observed in other NTDs: killed *Schistosoma mansoni* cercariae and irradiated parasites elicited strong immunity in animal models, but translation to humans was hindered by biosafety and production feasibility (13). These experiences underscored the trade-off between immunogenicity and safety in pathogen-based vaccines.

### 2.2 Second-generation vaccines: subunit and toxoid formulations

Second-generation vaccines, based on purified antigens or toxoids, improved safety and standardization. Examples include the hepatitis B surface antigen and tetanus toxoid, both highly effective when paired with suitable adjuvants (14). However, these formulations are generally less immunogenic against pathogens that require strong cell-mediated immunity. In leishmaniasis, extensive efforts have focused on recombinant protein vaccines. The Leish-F1 polyprotein (a fusion of TSA, LmSTI1, and LeIF) combined with Monophosphoryl Lipid A (MPL) in an oil-in-water emulsion system (MPL-SE) adjuvant advanced to phase II trials, demonstrating induction of Th1 responses (e.g., IFN-γ, IL-12) in healthy volunteers and individuals from endemic regions (15). The more recent Leish-F3+ (including NH36 from *L. donovani*) formulated with glucopyranosyl lipid adjuvant (GLA) formulated in a stable emulsion (GLA-SE) showed safety and immunogenicity in clinical testing (16). Despite these advances, efficacy in preventing infection or disease progression has been inconsistent, reflecting the difficulty of maintaining strong CD8^+^ T cell activation with protein antigens alone (17). Comparable strategies in other NTDs illustrate similar limitations. The Sm14 fatty acid-binding protein subunit vaccine for schistosomiasis has advanced to phase II clinical trials in Brazil and Africa (18). In Chagas disease, subunit vaccines based on trans-sialidase and Tc24 proteins have entered preclinical development (19). While safe and immunogenic, these formulations often require potent adjuvants and optimized delivery systems to achieve protective efficacy. Second-generation vaccines provide a controlled and scalable alternative but highlight the challenge of breaking immune tolerance, preventing immune deviation, and inducing durable Th1/cytotoxic T lymphocyte (CTL) responses critical for controlling intracellular protozoans like *Leishmania*.

### 2.3 Third-generation vaccines: DNA and recombinant viral vectors

The advent of molecular vaccinology enabled antigen delivery through genetic material. DNA vaccines involve direct injection of plasmids encoding target antigens, leading to endogenous expression, antigen processing via both MHC-I and MHC-II pathways, and the induction of humoral and cellular responses (20). In murine models of leishmaniasis, DNA vaccines encoding LACK, gp63, or kinetoplastid membrane proteins provided significant protection, associated with IFN-γ production and parasite clearance (21). Canine studies also demonstrated immunogenicity, supporting translational relevance (22). Despite their promise, DNA vaccines generally suffer from low immunogenicity in humans, necessitating strategies such as electroporation or heterologous prime-boost regimens (23).

Recombinant viral vectors (adenoviruses, modified vaccinia Ankara [MVA], and others) offer higher transduction efficiency and strong innate immune activation. They are particularly effective in generating CTL responses, critical for clearance of intracellular pathogens (24). In leishmaniasis, adenoviral vectors encoding HASPB or KMP11 have been evaluated in preclinical models, showing induction of effector T cell responses and partial protection (25). The ChAd63-KH vaccine (chimpanzee adenovirus - ChAd63 - vector to deliver KMP-11 and HASPB) progressed to phase IIa trials, demonstrating safety and robust CD8^+^ T cell induction in humans (26, 27), however, in the recent randomized double-blind phase IIb trial in post–kala-azar dermal leishmaniasis (PKDL), a single vaccination did not achieve significant therapeutic efficacy in terms of clinical improvement or disease regression (28). This disparity between the success in safety and immunogenicity trials and the failure in the therapy against PKDL suggests that the prophylactic success does not guarantee a therapeutic success with the same third generation vaccine and that VL and PKDL may indeed be different diseases and may not necessarily by the same third generation vaccine, engendering the need for a GenIV vaccine.

Comparable advances in other NTDs include adenovirus- and MVA-based vaccines targeting *Schistosoma* and *Trypanosoma cruzi*, which have shown efficacy in animal models (29). Challenges remain in scaling production, addressing vector-specific immunity, and ensuring long-term safety. Nevertheless, third-generation platforms provide a rational pathway to overcome the limitations of subunit vaccines.

### 2.4 Fourth-generation vaccines: from molecular design to emerging applications

The most recent revolution in vaccinology is driven by synthetic nucleic acids and nanoparticle engineering. These fourth-generation platforms represent a fundamental shift from traditional antigen delivery toward precision molecular design capable of stimulating both innate and adaptive immunity. mRNA vaccines, in particular, activate innate immune sensors through RNA motifs and induce robust Th1 and CD8^+^ T cell responses, while nanoparticle formulations enhance antigen stability, trafficking, and immune presentation (30).

In the context of NTDs, early preclinical studies are promising. mRNA vaccines encoding fusion antigens such as PpSP15-LmSTI1 or other conserved antigens have induced multifunctional Th1-biased responses and reduced parasite burdens in murine models (31). The modular nature of mRNA technology enables rapid redesign to target different *Leishmania* species or antigens, a key advantage given the zoonotic diversity and regional variation of leishmaniasis. Nanoparticle-based vaccines extend this paradigm by organizing antigens into virus-like particles (VLPs), protein scaffolds, or synthetic carriers that mimic pathogen-associated structures. Such multivalent presentation enhances B cell activation, germinal center formation, and T cell priming (32). For NTDs, nanoparticles can encapsulate *Leishmania* proteins or peptides alongside immunostimulatory molecules, such as TLR agonists, improving delivery to dendritic cells and amplifying Th1 polarization (32, 33). Similar strategies are under development for schistosomiasis and hookworm infections, where nanoparticle formulations have enhanced antigen presentation and immune protection in preclinical models (34, 35). The adaptability of GenIV platforms is also relevant for pathogens with high antigenic diversity or multi-stage life cycles (e.g., *Plasmodium* spp.) and for arboviruses such as dengue, where multivalent designs may be required to broaden protection. Collectively, these findings demonstrate that GenIV platforms integrate safety, adaptability, and immunogenic potency features long sought in the development of vaccines against complex eukaryotic pathogens.

Beyond NTDs, the transformative potential of GenIV vaccines lies in their versatility and adaptability across distinct disease categories. These platforms, most prominently mRNA and advanced nanoparticle formulations, transcend the boundaries of classical prophylactic vaccination, expanding into therapeutic immunology and precision medicine. The unprecedented event of mRNA vaccines during the COVID-19 pandemic catalyzed rapid innovation and investment, accelerating translation into multiple infectious and non-infectious indications (4). Their capacity for rapid design, scalable production, and efficient immune induction makes these platforms particularly suited for pathogens with high antigenic variability or epidemic potential. Candidate mRNA vaccines have been developed for influenza, wherein seasonal antigenic drift and pandemic potential demand frequent reformulation. The ability to update sequences within weeks offers a major advantage over egg- or cell-based platforms (36). Similarly, investigational mRNA vaccines for Zika virus and cytomegalovirus (CMV) have entered clinical testing, demonstrating robust immunogenicity and favorable safety profiles (37, 38). For malaria, nucleic acid vaccines encoding circumsporozoite protein (CSP) or thrombospondin-related adhesion protein (TRAP) are in preclinical or early clinical development, aiming to overcome the limited efficacy of current subunit vaccines (39, 40). The flexibility of these platforms is especially relevant for pathogens such as HIV and *M. tuberculosis*, where antibody responses alone are insufficient for protection. In HIV, mRNA vaccines encoding stabilized Env trimers or mosaic antigens are being evaluated for their ability to induce broadly neutralizing antibodies and strong T-cell immunity (41, 42). In tuberculosis, mRNA constructs encoding multiple *M. tuberculosis* antigens have elicited protective Th1 responses in murine models, and several candidates are progressing to first-in-human trials (43). Dengue virus (DENV) is another important case where next-generation vaccine approaches remain highly relevant. The presence of four serotypes and the risk of antibody-dependent enhancement (ADE) complicate vaccine design, requiring balanced and durable immunity across serotypes. GenIV platforms may provide advantages by enabling rapid redesign of multivalent constructs, improved antigen stabilization, and flexible boosting strategies (44–46). Experimental mRNA vaccines encoding stabilized membrane and envelop structural or non-structural protein antigens from multiple DENV serotypes have shown the capacity to induce broadly neutralizing antibody responses in preclinical models, while minimizing imbalanced serotype dominance (47–51). These nanoparticle-based formulations displaying recombinant E protein domains or virus-like particle architectures have demonstrated improved antigen presentation and immune focusing in experimental settings. Although clinical translation remains challenging, experimental nucleic acid and nanoparticle-based dengue vaccines may help address limitations of earlier approaches and broaden protection in endemic regions.

**Figure 1:**
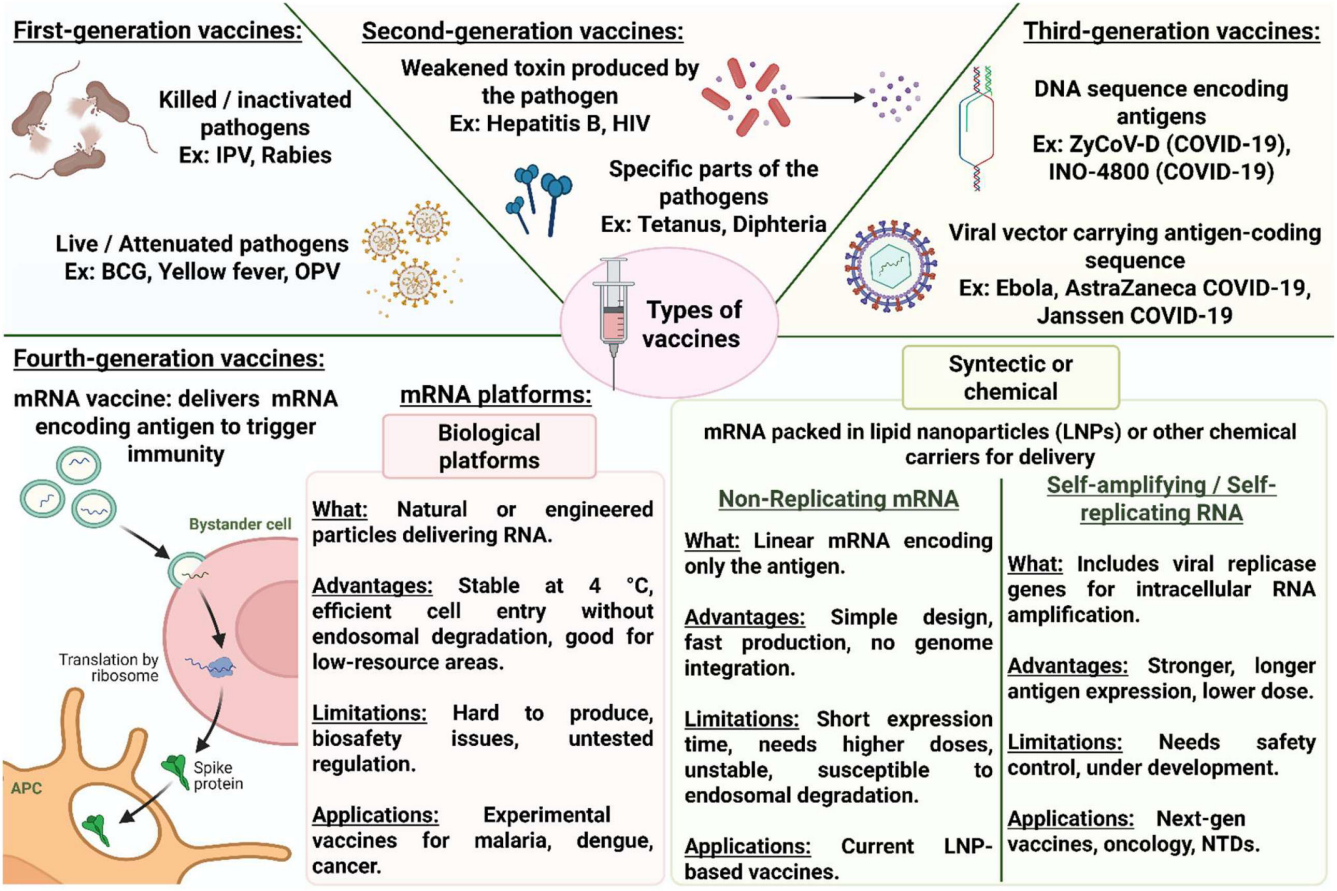
Evolution of vaccine generations and the emergence of mRNA platforms. Vaccines have evolved through four generations, defined by their antigen source and delivery strategy. First- and second-generation vaccines rely on whole pathogens or purified antigens, respectively, while third-generation vaccines use DNA or viral vectors encoding antigenic sequences. Fourth-generation vaccines, based on messenger RNA (employ synthetic lipid nanoparticle (LNP)-based systems or biologically inspired carriers such as viral vectors, virus-like particles (VLPs), and extracellular vesicles (EVs). These synthetic systems offer high biosafety and flexibility but still face challenges in stability and large-scale manufacturing. IPV, Inactivated Poliovirus Vaccine; BCG, Bacillus Calmette–Guérin (tuberculosis vaccine); OPV, Oral Poliovirus Vaccine; HIV, Human Immunodeficiency Virus; APC, Antigen-Presenting Cell.

The breadth of emerging applications illustrates a fundamental redefinition of vaccinology. GenIV vaccine platforms unite the strengths of molecular precision, immunogenic potency, and manufacturing flexibility, enabling rapid adaptation to new or re-emerging pathogens. Their convergence with genomics, nanotechnology, and synthetic biology opens opportunities that extend beyond infectious-disease prevention to therapeutic cancer vaccines, autoimmune modulation, and even personalized immunotherapy (**Figure 1**). However, realizing these benefits for NTDs will depend on delivery, manufacturing, and regulatory readiness discussed in the next sections.

## 3 Technological innovations in fourth-generation platforms

Building on the immunological rationale outlined above, this section addresses the technological and engineering aspects of GenIV, with particular emphasis on RNA design, delivery systems, and manufacturing-relevant innovations. Advances in mRNA vaccine technology paved the way for increasingly effective and adaptable immunization strategies. RNA platforms offer a superior biosafety profile. Whereas DNA molecules carry a non-zero probability of integrating into host genomes through illegitimate recombination, raising potential genotoxicity concerns, RNA molecules cannot integrate and therefore avoid this risk.

Current mRNA platforms can be divided into two categories: synthetic or chemical platforms such as Lipid NanoParticles (LNPs) and biological platforms such as viral vectors, virus-like particles or extracellular vesicles (EVs). Chemical platforms are the most advanced and clinically validated form of RNA delivery. Three LNP-based mRNA vaccines have received regulatory approval in the United States and the European Union, all targeting SARS-CoV-2. These systems provide high biosafety and potent immunogenicity but still face limitations in thermal stability at ambient and physiological conditions. Cellular uptake can also be constrained by endosomal degradation of the internalized mRNA (52). Ongoing optimization efforts aim to enhance both stability and endosome escape, primarily through chemical modifications of RNA bases. However, these modifications may inadvertently impair translation efficiency or elicit undesired inflammatory responses (53).

Beyond linear, non-replicating mRNA, self-amplifying RNA (saRNA) and self-replicating RNA (srRNA) platforms have emerged as promising innovations bridging the gap between conventional chemical vectors and biological systems. Derived from positive-sense RNA viruses such as alphaviruses, these constructs include a replication module encoding viral non-structural proteins that mediate intracellular RNA amplification (54). This design allows a stronger and more sustained antigen expression, often from markedly lower doses than conventional mRNA vaccines (55). Nonetheless, careful biosecurity measures are required to ensure that self-amplification remains tightly controlled and non-toxic (56).

Biological RNA delivery systems remain less mature, with no licensed products to date yet they hold important advantages in efficacy, stability, and design flexibility. Biological particles such as viral vectors, VLPs, or extracellular vesicles are inherently more stable under ambient conditions, a critical requirement for vaccine deployment in low-resource settings or tropical regions disproportionately affected by NTDs. Their mechanisms of cell entry are also more efficient than those of synthetic vectors and are not limited by endosomal sequestration or degradation (57).

Despite these advantages, biosafety and manufacturability remain key challenges. mRNA viral vectors may raise biosafety concerns due to residual replication potential, whereas extracellular vesicles offer improved safety but are limited by production yield and difficulties in quality control (58). A promising future direction may involve chimeric vesicles, which combine the biosafety of exosomes with the efficiency, scalability, and controllability of viral vectors. Taken together, these platform innovations provide a strong technical basis for GenIV vaccine development; however, their real-world impact on NTDs will ultimately depend on manufacturing readiness, regulatory alignment, and equitable implementation strategies discussed below.

## 4 Regulatory, manufacturing, and delivery challenges

The rapid evolution of mRNA technologies over recent years has revealed both the transformative potential and the complex challenges of RNA-based vaccines. Lessons learned from the development and deployment of SARS-CoV-2 vaccines have provided a foundation for streamlining regulatory pathways, improving manufacturing scalability, and expanding applications beyond pandemic response. However, to fully realize the promise of GenIVs, particularly for neglected and endemic diseases, several regulatory, industrial, and logistical barriers must be addressed.

Chemical platforms such as lipids nanoparticles–based mRNA vaccines benefit from an established regulatory and industrial framework. Large-scale production processes have been validated, costs are better characterized, and safety and efficacy profiles are well documented through COVID-19 experience. Yet, several constraints remain. Manufacturing costs remain high, and cold-chain requirements continue to limit deployment in low-resource regions. Improving thermostability and lowering production costs are essential to enable widespread use in developing countries, where affordability and heat resilience are key determinants of vaccine accessibility.

Recent global initiatives provide concrete examples of how GenIV vaccine manufacturing can be decentralized and adapted to low- and middle-income country (LMIC) contexts (59). Notably, the WHO has established a global mRNA technology transfer hub in South Africa, coordinated by Afrigen Biologics and supported by regional partners such as Biovac, with the aim of enabling local production of mRNA vaccines through open-access technology transfer and workforce training (*The MRNA Vaccine Technology Transfer Hub: A Pilot for Transformative Change for the Common Good?*, n.d.). This initiative has already facilitated the development of locally produced mRNA vaccine candidates and serves as a blueprint for regional manufacturing ecosystems beyond pandemic settings. In parallel, several LMIC-based manufacturing initiatives are expanding capacity for genetic and nanoparticle-based vaccines. The WHO’s mRNA Technology Transfer Hub in South Africa, with partners across regions including Brazil, India, Indonesia, and Senegal, exemplifies coordinated efforts to transfer mRNA production know-how and build local manufacturing and regulatory capacity. In Africa, multiple countries (e.g., Senegal) have been selected to receive technology for local mRNA vaccine production, representing concrete steps toward decentralized manufacturing. While traditional vaccine production capacity (e.g., in India) has historically been strong, emerging collaborations, such as evaluations by global partners to establish sustainable mRNA manufacturing facilities in Rwanda and Senegal, further underscore this trend (60; *The MRNA Vaccine Technology Transfer Hub: A Pilot for Transformative Change for the Common Good?*, n.d.). Collectively, these initiatives highlight how GenIV platforms, owing to their synthetic and modular nature, are particularly well suited for decentralized manufacturing models, reducing reliance on high-income country supply chains and improving responsiveness to regional infectious disease priorities.

Biological RNA vectors, including virus-like particles, viral vectors, and extracellular vesicles, face a different set of challenges. Although these platforms offer superior intrinsic stability, often maintaining efficacy at 4 °C and tolerating several days at ambient temperature, their industrial scalability, biosafety parameters, and regulatory pathways remain largely untested. Production yields and cost-effectiveness are not yet defined, and quality control standards for complex biological particles have yet to be harmonized. Establishing standardized evaluation pipelines will be critical before large-scale deployment can be considered.

From a technological perspective, the future of RNA vaccination lies in the integration of chemical and biological design principles. Hybrid or chimeric systems, merging the biosafety and modularity of exosomes with the efficiency and scalability of viral or lipid-based vectors, could provide unprecedented flexibility. At the same time, innovations such as self-amplifying and circular RNA constructs may enhance antigen expression, prolong immune responses, and reduce the RNA dose required per vaccination. These approaches could simplify the manufacturing workflows and lower production costs, directly addressing current bottlenecks in global deployment.

On the manufacturing side, advances in continuous and cell-free bioprocessing, standardized quality-control pipelines, and lyophilization technologies could enable the development of thermostable, ready-to-use formulations that no longer depend on ultra-cold storage. Such progress would be transformative for low- and middle-income countries, where logistics, infrastructure, and cost constraints have historically limited access to advanced vaccines.

From a regulatory standpoint, international collaboration will be crucial to harmonize standards for RNA-based products, including biological vectors. The creation of predictive safety models, standardized potency assays, and mutual recognition agreements between agencies could accelerate clinical translation while maintaining safety and quality standards.

Finally, from a global health perspective, RNA platforms hold the potential to revolutionize vaccine equity. Their modularity enables rapid adaptation to newly emerging pathogens, while localized manufacturing networks—based on flexible RNA synthesis and formulation hubs—could empower regional production capacities in Africa, Asia, and Latin America. In this vision, mRNA technologies would not only serve as a response to pandemics but also as a sustainable tool to combat neglected and endemic diseases, including malaria, dengue, and leishmaniasis.

## 5 From innovation to equity: fourth-generation vaccines for neglected tropical diseases

NTDs continue to lack licensed human vaccines due to a combination of scientific complexity, limited market incentives, and infrastructural constraints in endemic regions, despite causing significant morbidity and mortality in endemic regions (10, 61). The immunological and technological properties of GenIV platforms are described in the previous sections. Here, we focus specifically on the translational and equity-related barriers that determine whether GenIV vaccines can realistically reach NTD-endemic populations, including cost, cold-chain feasibility, manufacturing decentralization, and regulatory harmonization.

Parasites like *Leishmania* present unique challenges, including antigenic diversity and complex life cycles involving multiple developmental stages in both insect vectors and mammalian hosts (62). Protective immunity often depends on robust and durable cell-mediated immune responses rather than antibody alone, necessitating vaccine platforms capable of eliciting multifaceted immune activation. Traditional vaccine approaches have struggled to meet these demands, emphasizing the critical need for innovative platforms (59).

GenIV platforms offer several advantages for tackling these challenges. Their capacity to encode multiple antigens and immunostimulatory molecules makes them well-suited for pathogens that require multifaceted immune responses. Unlike conventional vaccines that often require culturing live or attenuated pathogens, synthetic production of nucleic acid vaccines eliminates biosafety concerns linked to handling live parasites and ensures consistent quality and scalability. For NTDs, the modular design of GenIV vaccine platforms can accelerate the identification and combination of protective antigenic targets while enabling region-specific vaccine updates (63).

However, technical innovation alone is insufficient to ensure that the benefits of these vaccines reach the populations that need them most. Systemic and infrastructural barriers in endemic regions, such as limited health infrastructure, insufficient cold chain logistics, and a lack of local manufacturing capacity, can severely hinder vaccine access. Investments in technology transfer, regional manufacturing hubs, and regulatory harmonization are therefore essential to support sustainable vaccine availability. Developing thermostable formulations and needle-free delivery methods further enhances feasibility in resource-limited settings by simplifying storage and administration (64). These considerations also apply to arboviral NTD-relevant infections such as dengue, where high disease burden, outbreak dynamics, and infrastructure limitations make deployability and regional manufacturing capacity central to vaccine impact.

Equity in vaccine distribution requires ensuring that all populations, particularly the most vulnerable, have fair and timely access to vaccines (65). Achieving this fairness involves addressing ethical and social considerations, including vaccine prioritization, which can expose structural inequalities in procurement and allocation and potentially exacerbate health disparities. High production costs and logistical complexity may limit availability to wealthier populations or urban centers, leaving marginalized communities at greater risk. In a pandemic, vaccines are a public good, and the State has a duty to ensure fair and timely access without bias or discrimination. Financial limitations or insurance status must never justify unequal treatment. Another challenge is unclear prioritization, which can lead to favoritism or queue jumping. Since vaccination programs are meant to protect those most at risk, bypassing this order delays access for vulnerable groups and heightens their risk of severe illness (66). This issue was particularly evident during the distribution of COVID-19 vaccines, where inequities in access and unclear prioritization often left vulnerable groups at greater risk. During COVID-19 pandemic, media reports allege vaccine distribution favored younger individuals, non-frontline government employees, and possibly elites, with government doctors prioritized over private practitioners despite both serving the public; however, queue jumping may be justified for those at higher risk of infection (67). At the center of these issues is the principle that the right to health is a fundamental human right, demanding equity, transparency, and accountability in vaccine allocation.

**Figure 2:**
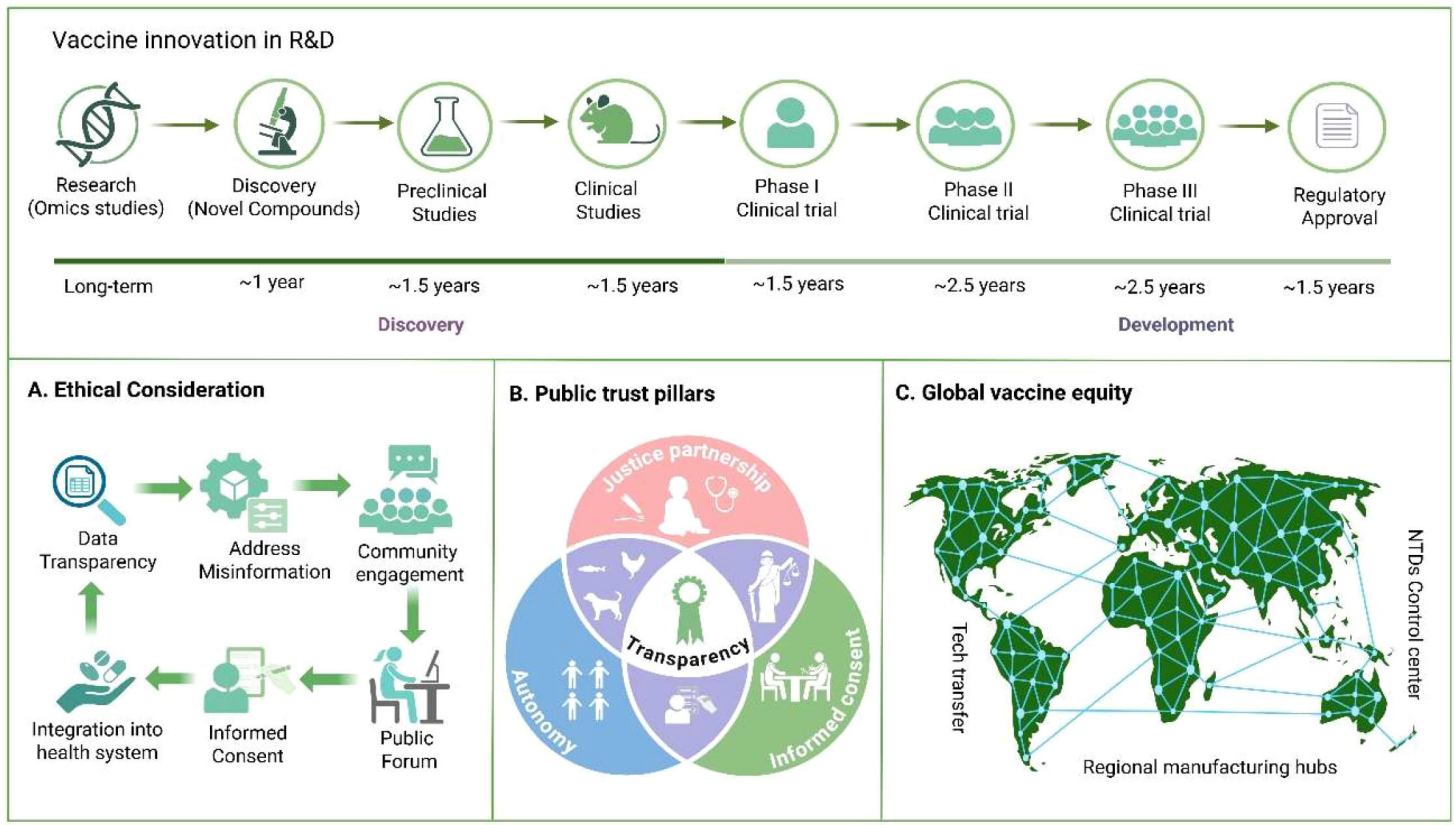
Vaccine innovation, ethics, and global equity framework. (Top) Vaccine R&D pipeline: spanning discovery (omics studies, novel compounds, preclinical and clinical studies) and development (Phases I–III clinical trials, regulatory approval), typically taking over a decade. **(A)** Ethical considerations: transparency, addressing misinformation, community engagement, informed consent, and integration into health systems. **(B)** Public trust pillars: justice partnership, autonomy, and informed consent, with transparency at the core. **(C)** Global vaccine equity: enabled through technology transfer, regional manufacturing hubs, and NTD control centers.

A global perspective is critical when considering the ethical deployment of GenIV vaccines. The COVID-19 pandemic revealed stark inequities in access to mRNA vaccines, emphasizing the need for frameworks that promote equitable distribution, international cooperation, technology transfer, and capacity building in low- and middle-income countries (68). Establishing regional manufacturing hubs, sharing technical knowledge, and reducing barriers to access are necessary steps to ensure that the benefits of genetic vaccine technologies extend beyond high-income nations (69). Finally, global collaboration, political commitment, and community engagement will be critical to realize the potential of GenIV for NTDs (70). Leveraging lessons from the rapid and large-scale deployment of COVID-19 mRNA vaccines, such as accelerated clinical trials and public-private partnerships, could catalyze similar breakthroughs for neglected diseases (71). GenIV vaccines hold unprecedented promise for addressing the unmet challenge of NTDs by combining scientific innovation with strategies aimed at global equity. Concerted efforts spanning basic research, infrastructure development, regulatory frameworks, and community-centered approaches will be critical to translate these technological advances into tangible health benefits for the world’s most vulnerable populations (**Figure 2**).

## 6 Bioethics and public trust in GenIV vaccines

The rapid development and deployment of GenIV vaccines, particularly mRNA and DNA-based platforms, has revolutionized modern immunization strategies while raising ethical concerns and challenges related to public trust (72). These challenges are especially relevant for GenIV vaccines, whose novelty, rapid development timelines, and perceived technological complexity can fuel uncertainty, misinformation, and fears of exploitation. While such platforms enable unprecedented responsiveness to emerging and NTDs, their successful deployment depends critically on public confidence and societal acceptance. These uncertainties are closely linked to broader social dynamics, where opposition rooted in religious beliefs, political ideology, or health concerns has fueled vaccine hesitancy (73). While a vaccine may not be fully effective, high population coverage remains essential to achieve herd immunity, thereby substantially protecting those who cannot be vaccinated or for whom the vaccine is less protective.

In NTD-endemic and resource-limited settings, ethical concerns surrounding GenIV vaccines extend beyond generalized vaccine hesitancy. They are shaped by misconceptions about nucleic acid technologies, often conflating genetic vaccination with gene editing, as well as by historical medical mistrust linked to structural inequities, limited local manufacturing, and delayed access to biomedical innovations (74, 75). These dynamics can undermine acceptance even when vaccines demonstrate strong safety and efficacy profiles. Transparent scientific communication emphasizing the transient nature of mRNA, its lack of genomic integration, and the robustness of preclinical and clinical evaluation is therefore essential but insufficient on its own (76). Ethical acceptability is also closely intertwined with issues of equity and access discussed earlier in this review. Dependence on external supply chains, inequitable allocation, and delayed regulatory approval can reinforce perceptions of injustice and erode trust in GenIV vaccine initiatives. From a bioethical perspective, trust relies not only on scientific rigor but also on fairness in distribution, transparency in decision-making, and respect for individual autonomy through meaningful informed consent (77).

Additional ethical challenges emerge in personalized vaccines such as cancer immunotherapy, where large-scale genomic sequence and individualized vaccine design raise critical issues related to data privacy and governance. Safeguarding sensitive genetic information through robust data protection, restricted access, and transparent consent processes is essential to prevent discrimination or misuse and to maintain public confidence in genetic technologies.

Autonomy in supporting patients in making decisions that align with their values and preferences, ensuring their choices are both informed and voluntary, is also important. Informed consent is vital because patients must fully understand the experimental nature of these vaccines, the potential risks involved, and the limitations of anticipated outcomes. Moreover, transparency about how genetic data is used, stored, and potentially shared for research builds trust and empowers patients to make informed decisions (77). Safeguarding autonomy at the individual level addresses only one aspect of the challenge. Building robust public trust requires collective action that goes beyond sharing factual information and involves ongoing community engagement, tailored public education, and meaningful participation in vaccine-related decision-making. Education campaigns should consider local cultural and societal contexts and leverage trusted community leaders, patient advocacy groups, and healthcare providers to bridge gaps between scientific advancements and public acceptance. This approach is reinforced by transparent communication about vaccine development processes, clinical trial outcomes, side effects, and ongoing safety assessments, which strengthens ethical accountability and fosters societal confidence. Involving the public in ethical decision-making bodies and advisory panels further enhances legitimacy and trust. By fostering participatory dialogue, vaccine developers and health authorities can address fears, counter misinformation, and align vaccine deployment strategies with community values and needs, ultimately promoting higher vaccine acceptance and equitable access (78).

Ultimately, addressing bioethical challenges and fostering public trust are central to realizing the full potential of GenIV vaccines. Achieving this goal requires a multidimensional approach that combines public education, transparent communication, robust data governance, equitable access, and culturally sensitive community engagement. By aligning scientific innovation with ethical responsibility, GenIV vaccines can be transformed into safe, effective, and socially responsible interventions with global impact.

## 7 Future outlook and considerations

Importantly, emerging clinical data from 2024–2026 have refined earlier assumptions about mRNA vaccine performance. While initial expectations focused primarily on speed of development and strong short-term immunogenicity, recent studies have highlighted additional considerations, including the durability of immune responses, the impact of repeated boosting, and the role of innate immune activation in shaping long-term efficacy and reactogenicity (23, 79). These insights have informed ongoing optimization of RNA design, dosing strategies, and delivery systems, reinforcing the relevance of GenIV platforms while underscoring the need for disease-specific and context-dependent evaluation. The development of GenIV with in-built flexibility of antigen design and enhanced immunogenicity represents a paradigm shift in controlling and potentially eliminating NTDs, offering highly targeted, safe, and long-lasting immunity (63). Translating the Covid-19 success to neglected diseases will require a similar commitment to scientific innovation, funding, and international collaboration. The future research must therefore prioritize AI-assisted definition of clear immune correlates of protection and robust biomarkers while minimizing off-target effects. Adapting clinical trial designs to overcome logistical challenges in resource-limited and endemic settings will be pivotal.

Integration with ongoing public health interventions, including mass vaccine administration, vector control, and sanitation initiatives, and rigorous evaluation of safety and immunogenicity across diverse populations will be crucial to maximize the impact of vaccination campaigns (80). Equitable access and cost-effectiveness remain central considerations, requiring coordinated efforts from governments, non-profit organizations, and public-private partnerships to facilitate global deployment (70). Expanding manufacturing capacity in low and middle-income countries through technology transfer, infrastructure investment, thermostable formulations, and needle-free delivery systems for vaccination is critical to ensure equitable vaccine access (69).

Beyond manufacturing and logistics, integrating vaccine development into broader One Health strategies offers particular promise for zoonotic NTDs such as leishmaniasis. Veterinary vaccines like Leishmune and CaniLeish have demonstrated reductions in transmission from animal reservoirs by interrupting zoonotic cycles, highlighting the potential of coordinated human-animal vaccination campaigns (81). Regional mRNA production hubs emerging in Africa, Latin America, and Asia provide practical models for building local capacity tailored to endemic needs and enhancing regional self-reliance, and allowing vaccine optimization for local populations (82). By aligning scientific innovation with infrastructure development, policy support, and community engagement, GenIV holds the potential to significantly advance the control, elimination, and eventual eradication of NTDs (10). In summary, the convergence of molecular innovation, scalable manufacturing, and equitable regulatory frameworks will define the future of RNA vaccines. Transitioning from emergency-driven production to sustainable, globally accessible platforms will mark the true legacy of the RNA revolution.
